# Effects of Urolithin A supplementation on performance and antioxidant status in academy soccer players during preseason: a pilot randomised controlled trial

**DOI:** 10.3389/fnut.2025.1674446

**Published:** 2025-10-30

**Authors:** Andrea Monsalve Acevedo, Colin Sanctuary, Robert John Aitken, Alex Wilkins, Natasha Harrison, Mitchell Naughton

**Affiliations:** ^1^School of Biomedical Sciences and Pharmacy, University of Newcastle, Callaghan, NSW, Australia; ^2^Applied Sports Science and Exercise Testing Laboratory, University of Newcastle, Ourimbah, NSW, Australia; ^3^Centre for Reproductive Science, University of Newcastle, Callaghan, NSW, Australia; ^4^Hunter Medical Research Institute, New Lambton Heights, NSW, Australia

**Keywords:** polyphenols, supplement, aerobic capacity, anaerobic exercise, countermovement jump

## Abstract

**Background:**

Polyphenol-derived compounds, such as Urolithin A (UA), may exert beneficial effects to performance adaptations during periods of high training stress through several pathways including a reduction in an oxidative stress and improved mitochondrial function. At present, the benefits of UA supplementation have been observed predominantly in clinical and preclinical models. This pilot study aimed to investigate the effects of UA supplementation on performance outcomes, antioxidant status, and intervention feasibility and acceptability during a six-week preseason period in academy soccer players.

**Methods:**

Twenty male academy soccer players (age: 17.5 ± 1.0 years) were randomly assigned 1:1 to receive 1,000 mg/day of UA or an isocaloric taste-matched placebo which was given post-training over the course of a six-week training intervention in a single-blinded, parallel-group design. The intervention was delivered alongside the team’s preseason training from November to December 2024. Primary outcome was aerobic endurance (Yo-Yo Intermittent Recovery Test Level 1), with secondary outcomes including lower-limb strength and power metrics (Countermovement Jump), maximal sprinting speed, and salivary antioxidant capacity using the RoXsta™ System. Each of the primary and secondary outcomes were assessed pre and post the intervention. Feasibility and acceptability of the intervention along with dietary intake was assessed via individual questionnaire, pre and post intervention, while subjective stress-recovery status was assessed via questionnaire administered weekly over the duration of the intervention. Primary and secondary data were analysed using linear mixed effects models, with group (UA/placebo) × time (pre/post) interactions interpreted using estimated marginal means.

**Results:**

UA supplementation led to significantly greater improvements in Yo-Yo IRT1 performance compared to placebo (Δ = +239 m, 95% CI [20, 454 m], *p* = 0.048). For secondary outcomes, countermovement jump height also improved in the UA group relative to placebo (Δ = +3.33 cm, [0.88, 5.95 cm], *p* = 0.020). No group x time differences were observed in sprint speed, jump power, impulse, or eccentric duration, or saliva antioxidant assays. Antioxidant activity declined significantly over time in the placebo group but did not so in the UA group. Feasibility and acceptability questionnaire responses indicated the intervention as it was delivered was rated with high feasibility and acceptability.

**Conclusion:**

Six weeks of UA supplementation during preseason improved aerobic endurance and some measures of jump performance in elite academy soccer players, while preserving aspects of antioxidant status. Confidence limits on the primary and secondary findings were broad. These findings potentially support UA as a feasible and well-tolerated intervention in athletic populations, warranting further research in larger and well powered confirmatory trials (Clinical Trial No. ACTRN12624000959572).

## Introduction

Soccer (also known as football) is an intermittent team sport characterised by periods of high intensity locomotor actions interspersed with periods of lower intensity movement (1). From a physical performance perspective, soccer players are required to be able to sustain prolonged periods of submaximal activity such as walking and jogging while also producing frequent high intensity efforts such as sprints, accelerations, change of direction, and jumps which are essential for successful performance at competitive levels (2, 3). In addition to the highly technical demands of performance, the physical nature of training and matchplay requires players to have highly developed capacities across various physiological domains including aerobic power, anerobic power and capacity, muscular strength, power, and agility (3, 4).

The preseason is a critical phase wherein concentrated training can occur to improve the physiological, technical, and tactical capabilities of players and teams. Depending on the player performance level and age group categorisation (i.e., junior, academy, or senior), preseason typically lasts 4–6 weeks during which athletes are exposed to progressively elevated training loads (5). This is achieved through a combination of increased volume and intensity (6). During this phase, the primary focus of training is on field and gym-based sessions and on improving the underlying physiology for performance—aerobic endurance, strength, speed and power. Elevated physiological stress and the intensified nature of training with increased training loads in preseason comes with inherent risks. These can occur, in part, due to the sharp increase in training volume and intensity, potentially impacting a player’s recovery capacity (7). From a physiological perspective, a key contributor to these issues is the accumulation of oxidative stress, which rises when the production of reactive oxygen species (ROS) increases, through muscle contractile activity and exceeds the physiological antioxidant defences, leading to cellular damage and impaired muscle recovery (8, 9). When insufficient recovery is provided, whether due to inadequate, nutrition, sleep, or periodisation errors (i.e., training sessions without sufficient intervening time), the effects of oxidative and metabolic stress can be exacerbated resulting in compromised training performance and increased injury risk (10).

During high-intensity training phases such as the preseason, there is a substantial energy expenditure increase, making it essential to strategically consume nutrients to support exercise and recovery. For example, consuming carbohydrates before and after training helps maintain or replenish glycogen stores (11). In addition to macronutrient focused nutrition, targeted supplementation interventions are frequently used to enhance athletic performance and minimise physiologically-related fatigue and oxidative damage. Recently, polyphenols and polyphenol-related compounds such as, tart cherry juice (12, 13), nitrate rich beetroot juice [e.g., Nyakayiru et al. (14)], and Urolithin A (15–17), have emerged as promising interventions due to their potential effects on oxidative stress, mitophagy, and inflammation.

Urolithin A (UA) is a naturally occurring metabolite produced by the gut microbiota through the transformation of dietary polyphenols known as ellagitannins, which are abundant in foods such as, pomegranates, raspberries, strawberries, and walnuts (18). The formation of UA in the human body is highly dependent on individual gut microbiome composition, with ~30% of people not producing UA (and UA metabolites) (19). Initial preclinical work identified UA supplementation as extending nematode lifespan significantly, whilst also improving running distance (~57%) and grip strength (~9%) in rodents compared to placebo control (20). This led to subsequent research in humans which observed supplementation of up to 1,000 mg/day to be safe and effective for inducing an upregulation in mitophagy which promoted favourable molecular signals and metabolic outcomes in older adults (15). Subsequent research by Singh et al. (16) identified 4 months of 1,000 mg/day of UA supplementation improved hamstring muscle strength, aerobic endurance capacity (i.e., VO_2peak_), and C-reactive protein, a key marker of inflammation, in older adults to a greater extent than placebo. Similar improvements in muscular endurance have been observed elsewhere (21). UA is purported to exert its beneficial effects through mitophagy induction, with potential antioxidant and anti-inflammatory effects reducing the burden of reactive oxygen species (ROS) and suppressing inflammatory cytokine pathways that are often elevated during periods of heavy exercise or injury (15).

Despite the potential for UA to improve outcomes, studies to date have predominantly focused on clinical and preclinical models, with only one recently published study having focused on athletic populations. Here, Zhao et al. (22) undertook a blinded, placebo-controlled study with 1,000 mg/day supplementation or placebo for 8 weeks with 20 weightlifters and examined a range of performance and health related markers before and after the intervention. This study identified UA supplementation, compared to placebo, led to improvements in maximal voluntary isometric contractions of the knee extensors (~8%), and bench press repetitions to failure with a 60% 1RM submaximal load (~11%) (22). Collectively, the available research suggests UA may improve outcomes which are potentially relevant to performance for athletic populations. However, original research investigating UA supplementation in supporting adaptations in sporting and athletic populations is currently limited and necessary to undertake to examine UA’s potential benefits.

The primary aim of the current pilot study was therefore to examine the influence of UA supplementation against a placebo control group in improving performance-related outcomes such as, aerobic endurance, strength and power output, and maximal sprinting speed over the course of a six-week preseason in academy soccer athletes. Secondly, we aimed to determine the influence of UA on improving antioxidant defence through the quantification of the ROS scavenging potential of saliva biofluid. Finally, as this was the first study to examine UA in this population, assessing the feasibility and acceptability of the intervention was the final aim of this study.

## Methods

### Ethical approval and consent

All players and their guardians were fully informed of the study’s aims, procedures, and potential risks before providing consent. Written informed consent was obtained from players aged 18 years and over and for players under 18 years of age, parental or legal guardian consent was obtained. The study was conducted in accordance with the Declaration of Helsinki and received ethical approval from the relevant institutional Human Research Ethics Committee (H-2024-0236) prior to commencement. This study was registered on 7/08/2024 as a clinical trial with the Australian and New Zealand Clinical Trials Register (ANZCTR; ACTRN12624000959572).

### Study design

This study employed a randomised, single-blinded, placebo-controlled design to investigate the effects of UA supplementation on performance parameters in sub-elite soccer players during a six-week preseason training period at the team’s training facility from November 2024 to December 2024. This was a parallel group trial where allocation occurred on a 1:1 basis. Following recruitment and pretesting, players were randomly allocated to either to receive UA supplementation or the placebo using a computer-generated randomisation sequence^1^ with a block size of four. This was conducted by co-author MN and other researchers were blinded to the allocation phase. Recruited players were blinded to group assignment to minimise potential bias whilst the researchers administering the trial were not blinded. The intervention was carried out concurrently with the players’ structured preseason training programme, allowing for the evaluation of UA’s effects under ecologically valid training conditions.

### Participants

Participants (*n* = 20, 17.5 ± 1 years of age, 178.1 ± 6.5 cm, and 74.3 ± 8.7 kg) were recruited from the same A-League Under 23 Academy team. At the commencement of the study, players were provided with the requirements of the study as outlined in the participant information sheet and given the option of participating in the study. The players were actively involved in regular training and match play with the academy’s squad during the data collection period. Players who participated occupied a range of outfield positions and included goalkeepers ensuring a representative positional sample. The number of participants allocated to the supplement and placebo interventions, assessed at follow-up, and included in the final analysis for the primary outcome measure is outlined in the CONSORT 2025 (45) diagram (Supplementary Figure 1).

Inclusion criteria required that players were currently registered with the academy, free from injury or illness, and medically cleared to engage in high-intensity training and testing activities. Players were excluded if they had sustained an injury in the 6 weeks prior to testing, were not regularly participating in training sessions, or had any known medical conditions that could increase the risk of adverse effects during physical exertion. This was determined by the team’s medical staff (i.e., team doctor or physiotherapist).

In relation to a player’s ability to participant in the training sessions, any player/s who sustained injuries during the preseason were withdrawn from active training sessions but continued to receive their assigned intervention (UA or placebo) through the remainder of the study period. Once players were deemed fit by the teams medical and performance staff they were subsequently returned to training.

### Training programme

All players followed a structured six-week preseason training programme designed and delivered by the club’s coaching and performance staff. The programme aimed to progressively develop physical qualities relevant to match performance, including, endurance, strength, speed, and power. The design and scheduling of the preseason training loads were carried out in accordance with previous research in football by Teixeira et al. (23) which had explored accumulated training and match loads in a similar cohort.

Briefly, the structured preseason programme included four to five field-based training sessions per week, which focused on, technical, tactical, and conditioning components, as well as three to four gym-based resistance training sessions. The overall training schedule was consistent across both the UA and placebo groups to ensure a uniform training stimulus, with the coaches blinded to group allocation.

### Training quantification

To quantify the field-based training loads, players wore the same 10 Hz GNSS device (Apex, STATSports, Ireland), which were worn during all on-field sessions with the device placed between the scapulae and fitted into a tight-fitting garment. In accordance with guidance from the manufacturer, the devices were turned on 10–15 min prior to each session and post session the devices were collected, and session data downloaded and stored on the teams athlete management software. Beato et al. (46) evaluated the validity and between unit variability of STATSports Apex 10 GNSS units for measuring distance and peak speed in team sports and observed high validity and low between unit variability with measurement errors ranging from ~1 to 2%.

The average satellite count for these sessions was 17.0 with the average horizontal dilution of precision (HDOP) being 0.4. Weekly locomotor workloads during the preseason period were determined using key variables: total distance (meters), high-speed running distance (>5.5 m/s; meters), sprint distance (>7.0 m/s; meters), and maximal sprint speed (km/h).

### Supplement

Players assigned to the UA group received 1,000 mg of flavoured Urolithin A powder (Mitopure, Timeline Nutrition, Switzerland) on each training occasion which was mixed into their post-training protein drink following each scheduled training session over the six-week preseason period. The protein drink contained 22.9 g of protein, 2.6 g of carbohydrates, and provided 491 kj of energy per serve. To provide a fat-soluble matrix, the drink was made with ~250 mL of full cream dairy milk which contained, 8.8 g of protein, 11.5 g of carbohydrates, 8.3 g of fat, and provided 650 kj of energy per serve. The supplement was administered under supervision of the researchers to ensure adherence and consistency in dosage.

Players in the placebo group received an isocaloric 1 g dose of a cherry-flavoured inert powder, also mixed with their post-training protein drinks made with the same full cream dairy milk containing an identical macronutrient mix as mentioned previously and provided in an identical manner to the UA group. The appearance, taste, and texture of the placebo (determined through piloting in the pretesting period by the researchers) was matched as closely as possible to the UA supplement to maintain blinding. All players, regardless of injury status, were instructed to consume their shakes within 30 min of completing training, and compliance was monitored by the research team after each session.

### Short recovery stress scale (SRSS)

Recovery stress states were monitored on the same training day of the week and on a weekly basis using the short recovery and stress scale (SRSS), a validated psychometric tool designed to assess athletes’ current levels of recovery and stress (24). The SRSS consists of eight subscales, with four assessing recovery (physical performance capability, mental performance capability, emotional balance, overall recovery) and four assessing stress (muscular stress, lack of activation, negative emotional state, overall stress). Each item is rated on a 7-point Likert scale ranging from 0 (does not apply at all) to 6 (fully applies).

Players were verbally and visually familiarised with the scale questions prior to testing and completed the SRSS at the beginning of every week on the day of weekly testing, and before any training was completed to avoid the influence of acute fatigue. They were instructed to complete the questionnaire independently and honestly without sharing their answers with the other players using an online survey platform (QuestionPro) interfaced with a digital tablet (iPad, Apple, Cupertino, CA, USA) to standardise the administration procedure.

The SRSS has previously demonstrated strong internal consistency and test–retest reliability for monitoring athlete wellbeing in applied settings (25), and allows for fluctuations in psychophysiological stress and recovery to be captured throughout the intervention period. Within this study, the overall recovery and overall stress subscales were included in the analysis.

### Supplement adherence and training monitoring

To ensure adherence to the supplementation protocol, all UA and placebo doses were administered by the research team and recorded in a customised spreadsheet. This spreadsheet documented the daily distribution of the supplement and placebo to track and ensure consistency across the study period. Other information, such as alterations to the players participation in the field session through, for example, minor injury, soreness, or being called up into train with the first team, were also recorded.

### Feasibility and acceptability questionnaire

To assess the feasibility and acceptability of the supplement and placebo interventions to the players, a questionnaire was developed which featured ratings of how feasible and acceptable the participants found the intervention as it was delivered in this study. This was a bespoke questionnaire which featured Likert rating scales and was designed for this study based on a previously validated intervention focused non-specific questionnaire by Sekhon et al. (26). Participants were familiarised with the questionnaire prior to data collection, and the questionnaire was administered to players at the end of the study protocol via the QuestionPro online survey platform.

### Dietary intake

To provide an assessment of the macronutrient and total energy consumption profile of the participants and how this changed over time, a dietary intake assessment was completed using a 24-h dietary recall method at the beginning and end of the six-week intervention. For this, participants were asked to detail all food and beverage items consumed during the previous 24 h, including portion sizes, preparation methods, and the timing and context of each eating occasion. Recalls were conducted using the online survey platform QuestionPro.

All dietary data were entered into FoodWorks (Xyris Software, Brisbane, Australia), which utilises the AUSNUT 2011–13 food composition database developed by Food Standards Australia New Zealand (FSANZ) to calculate nutrient intakes. From these data, daily energy intake (kJ/day and kcal/day), macronutrient distribution (total intake [g], and percentage energy from carbohydrates, total fats, and protein) were derived from analysis.

### Outcome measures

#### Performance assessments

Performance testing was conducted at the beginning of the preseason and again at the end of the six-week preseason period under standardised conditions to assess changes in performance parameters including, aerobic endurance capacity, lower limb strength and power, and maximal sprinting speed.

The primary performance outcome measure and assessment was that of aerobic endurance. This was assessed through the Yo-Yo Intermittent Recovery Test Level 1 (Yo-Yo IRT1) using the standardised methods originally described by Bangsbo et al. (27). Previous meta-analytic research has identified a strong relationship between YoYo IRT1 performance and lab-based VO_2max_ assessment (*r* = 0.65) (28). The test involves intermittent shuttle running activity between a set of cones of increasing intensity (velocity) until the participant reaches volitional exhaustion and terminates the test. Players performed the test with encouragement by the team staff, with total distance covered (in meters) recorded as the primary measure of aerobic endurance.

A secondary performance outcome was the measurement of lower limb strength and power output. This was assessed using the Countermovement Jump (CMJ) test performed on a dual force plate system (ForceDecks, VALD Performance, Brisbane, Australia, 1,000 Hz). Key variables measured and analysed included, jump height (impulse-momentum; cm), peak force (Newtons), concentric impulse (N·s_−1_), eccentric duration (msec) and peak power (Watts). These variables have shown a high level of validity and reliability in recent work by Collings et al. (29).

The final performance outcome measure assessed was that of speed and acceleration. Here, a 40-m sprint test, conducted on the academies regular training field, was assessed through the use of electronic timing gates (Smart Speed 3.7 V 10.4 Ah, Fusion Sport, Coopers Plains, Australia,). Timing gates were placed a 0 and 40 m, with a further set placed at 50 m which provided participants with encouragement to run through the final set of gates.

#### Saliva collection and antioxidant assay

Saliva biofluid was collected from the players via the passive drool technique into aliquots with saliva collected at the beginning of the preseason and again at the end of the six-week preseason period. Briefly, players were provided with a visual familiarisation of the passive drool collection technique and the SalivaBio Passive Drool Saliva Collection Aid (SCA) and 2 mL Cryovial aliqouts (Salimetrics, State College, PA, USA). Participants instructed to consume no fluid for 10 min prior to providing then sample and then provide 1 mL of saliva via passive drool as per the manufacturers guidelines with the SCA placed into their mouth. Following collection, samples were frozen immediately at −80 °C for analysis of antioxidant concentration assays *en bloc* using the RoXsta™ System (30) at the conclusion of the study.

The following assays were utilised to evaluate three distinct types of antioxidant activity in saliva using ABTS as a redox-sensitive indicator.

1. Organic Hydroperoxide Scavenging Activity, determined by measuring the inhibition of ABTS• + radical formation in the presence of cumene hydroperoxide and hematin. The reaction mixture contains 225 μL of phosphate buffer (pH 6.5), 8.3 μL 10 mM ABTS (final concentration 250 μM), 33.3 μL sample (20% dilution), and 33.3 μL hematin (0.05 mg/mL final concentration). The reaction was initiated by the addition of 33.3 μL 1 mM cumene hydroperoxide (final concentration 100 μM), followed by incubation at room temperature for 20 min. Absorbance was then measured at 734 nm using a spectrophotometer (SPECTROstar Nano, BMG Labtech).
2. Hydrogen Peroxide Scavenging Activity, to determine the samples’ ability to inhibit ABTS• + radical formation catalysed by hydrogen peroxide. The reaction mixture comprised of 245 μL phosphate buffer (pH 6.5), 5 μL of ABTS (150 μM final), 33.3 μL saliva (30% dilution), and 16.7 μL of horseradish peroxidase (HRP; 0.05 mg/mL final). After the addition of 33.3 μL 300 μM hydrogen peroxide (final concentration 30 μM) to initiate the reaction, the mixture was incubated for 10 min before measuring absorbance at 734 nm.
3. Free Radical Scavenging Activity, based on the oxidation of ABTS. In this assay, radicals are electrochemically generated by oxidising ABTS (100 μM) in phosphate buffer (pH 4.8) using an electrochemical cell. Following activation, 360 μL of the radical-containing solution was combined with 18 μL of saliva (50% dilution) and the reduction in absorbance recorded after a 5 min incubation.

For all antioxidant assays, ascorbic acid was utilised as a standard, with results expressed in terms of Vitamin C Equivalents (mM).

### Statistical analysis

The study was exploratory in nature to inform the development of future UA studies. Initially, variables were assessed by visually inspecting diagnostic plots, including quantile-quantile (Q-Q) plots to confirm normality of residuals, and residuals versus fitted values to confirm homoscedasticity. Thereafter, as these checks indicated these assumptions were met, to assess the effects of UA supplementation compared to placebo on performance changes through preseason, data was analysed using serial linear mixed effects models. This approach accounted for different sources of variability, missing data, and the pre/post design of the study. These linear mixed effects models included fixed effects for time (i.e., pre-, vs. post-intervention), group (i.e., UA vs. placebo), and the group x time interaction. The pre-intervention value was included in the model as a covariate, while random effects were included for each participant.

As the aim of this study was to assess the influence of UA or placebo on the training effects from pre- to post-intervention, the group x time interaction was the main model of interest and is the analytical focus of the results. Further, models were specified with the pre-intervention value and the placebo group set as the reference categories. This approach allowed all intervention and time effects to be interpreted directly from the model estimates. To confirm any differences within a treatment arm but between time points, pairs analysis is also presented for the placebo and UA-treatment group, respectively.

Data handling and analyses were conducted using R open-source programming language using the RStudio GUI. Packages used for this included *lme4* (31), *lme4test* (32), *tidyverse* (47), and *emmeans* (48). Results were visualised using Graphpad Prism for Windows (v10, GraphPad Software, Boston, MA, USA). Primary outcome and secondary outcome results are presented as bar charts with mean ± standard deviation (SD) with individual participant data overlayed for transparency (33). For all analyses, statistical significance was set *a priori* at *p* < 0.05, and *β* regression coefficients and 95% confidence intervals (CI’s) were reported for the effect sizes in the same units and scale of analysis to aid in interpretability and variability of the effect.

## Results

### Feasibility and acceptability questionnaire

The results of the feasibility and acceptability questionnaire for the intervention indicated that there was a generally positive response to the intervention as it was delivered across both the supplement and placebo groups.

Most players ‘liked’ or ‘strongly liked’ the supplement, with 65% selecting ‘like’ and 20% selecting ‘strongly like’. Exploring the effort to engage with the intervention, 60% reported it took ‘little effort’, and 25% said it took ‘no effort at all’. In terms of perceived effectiveness, here, 50% ‘agreed’ and 15% ‘strongly agreed’ that the intervention improved their aerobic endurance capacity. In addition, 55% ‘agreed’ it improved their force and power. Finally, 50% ‘agreed’ and 10% ‘strongly agreed’ that they understood how the UA supplement let to potential training adaptations. The feasibility of future implementation was also rated positively—55% ‘agreed’ and 30% ‘strongly agreed’ that it would be feasible to explore the UA supplement with a larger group. Lastly, 80% ‘disagreed’ or ‘strongly disagreed’ that the supplement interfered with other priorities. Plus, 60% found the intervention ‘acceptable’, with ‘20%’ rating it as ‘completely acceptable’.

The full summary for the feasibility and acceptability questionnaire can be found in Supplementary Table 1.

### Training and training quantification

Adherence to training, either by the completion of the full session or with minor modifications was 91.2 ± 7.6% in the UA group, and 75.6 ± 20.2% in the placebo group. Common reasons for absences from training included being called up to train or play with the senior men’s team, treatment for minor injury, illness, or rest. On these occasions, players were still at the training centre and consumed their allocated beverage.

The GNSS-derived training workload characteristics of the players from throughout the preseason period are presented below in Table 1. The average satellite count for these sessions was 17.0 with the average horizontal dilution of precision (HDOP) being 0.4.

**Table 1:** Weekly external load GNSS-derived metrics for the Urolithin A (UA) and placebo groups across the six-week preseason period for metrics including total distance, high-speed running (HSR), sprint distance, and max speed.

Metric	Group	Week 1	Week 2	Week 3	Week 4	Week 5	Week 6
Total distance (m)	UA	20,525 ± 1,009	26,677 ± 8,748	18,231 ± 7,694	24,380 ± 3,274	23,437 ± 6,673	22,083 ± 6,085
	Placebo	18,268 ± 6,546	24,875 ± 5,712	14,033 ± 7,551	19,555 ± 6,084	20,557 ± 7,186	21,268 ± 4,194
HSR distance (m)	UA	657.6 ± 138.8	1,260.4 ± 624.6	424.3 ± 121.4	952.0 ± 198.8	740.3 ± 316.2	895.9 ± 322.0
	Placebo	522.6 ± 291.8	970.6 ± 343.7	662.7 ± ± 401.0	819.8 ± 391.1	910.7 ± 509.5	722.2 ± 284.9
Sprint distance (m)	UA	40.4 ± 20.3	192.8 ± 172.1	48.6 ± 46.4	124.4 ± 60.4	48.8 ± 46.4	209.3 ± 58.4
	Placebo	34.5 ± 34.2	126.6 ± 83.3	74.6 ± 53.2	76.5 ± 36.4	173.1 ± 266.0	167.4 ± 46.4
Max speed (km/h)	UA	28.2 ± 1.0	30.7 ± 1.5	29.5 ± 1.7	30.4 ± 1.1	28.3 ± 1.5	33.4 ± 1.2
	Placebo	27.3 ± 2.3	29.5 ± 1.7	28.4 ± 4.5	28.6 ± 2.3	30.1 ± 4.3	32.6 ± 4.1

All values are mean ± standard deviation (SD).

NB – HSR distance = > 5.5 m/s, and sprint distance = > 7.0 m/s.

### Short recovery and stress scale

The week-to-week changes in SRSS Overall Stress and Overall Recovery subscales over the six-week preseason are presented below (Table 2).

**Table 2:** Weekly short recovery and stress scale (SRSS) overall stress and overall recovery subscale results.

Metric	Week 1	Week 2	Week 3	Week 4	Week 5	Week 6
Overall stress	2 ± 1	2 ± 1	2 ± 2.25	2 ± 2	2 ± 2	2 ± 1.5
Overall recovery	4 ± 2	4 ± 1.25	4 ± 1	4 ± 1.75	4 ± 1	4 ± 0

All values are median and interquartile range (IQR).

### Outcome measures

#### Performance assessments

**Figure 1:**
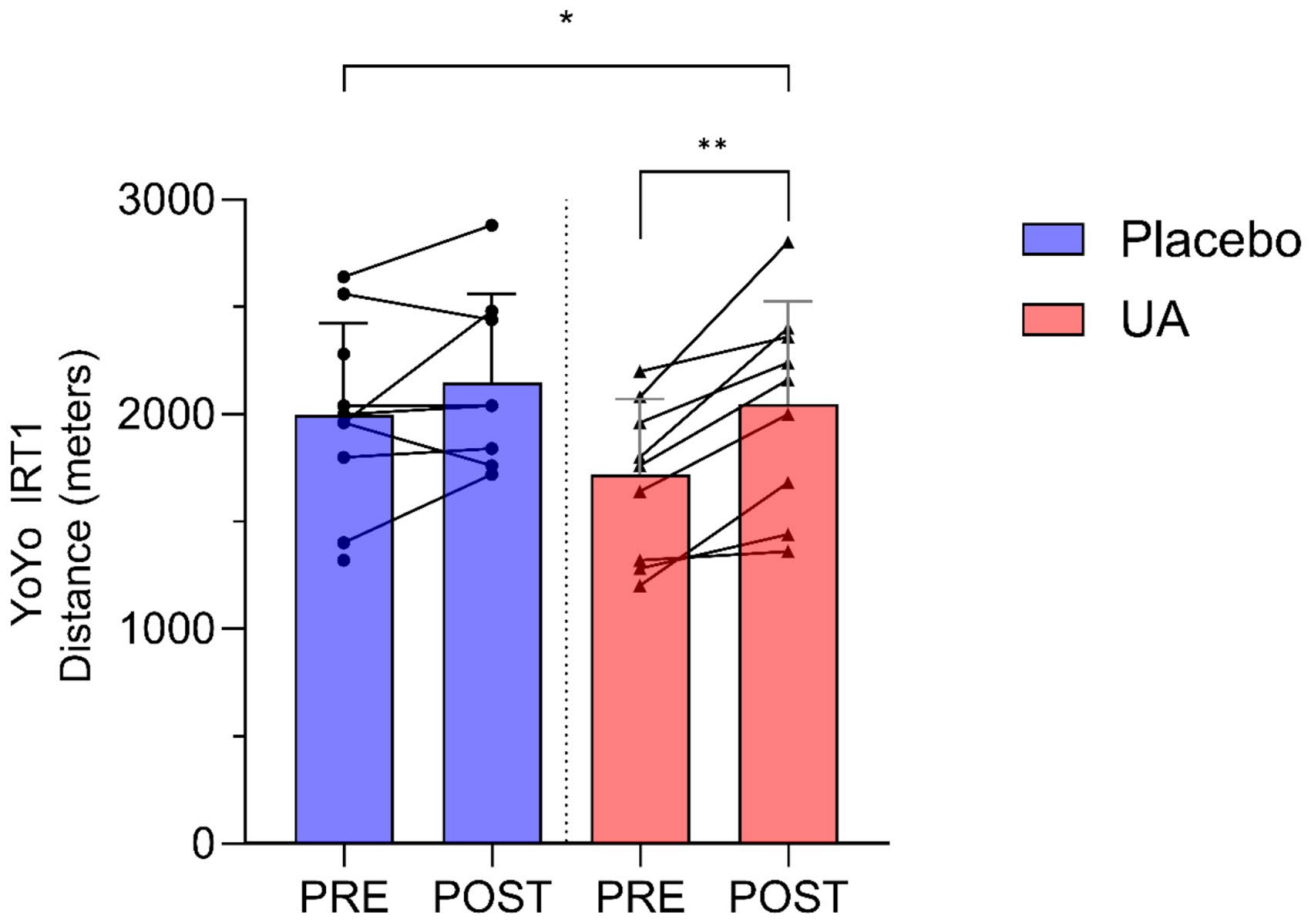
Results for Yo-Yo Intermittent Recovery Test Level 1 (Yo-Yo IRT1) distance (meters) pre and post intervention for Urolithin A (UA) and Placebo groups. Individual data points and lines represent individual players, and error bars denote standard deviation around each mean. *p < 0.05, **p < 0.01.

A significant interaction effect between group and time was found for the Yo-Yo IRT1, with the UA group demonstrated a significantly greater improvement in performance compared to those in the placebo group (*F*(1, 15.27) = 0.661, *p* = 0.048). The estimated increase (*β*) in performance for the UA group relative to placebo was 239 (95% CI [20, 454]) meters. Individual and group-level changes in Yo-Yo IRT1 performance are illustrated below (Figure 1).

**Figure 2:**
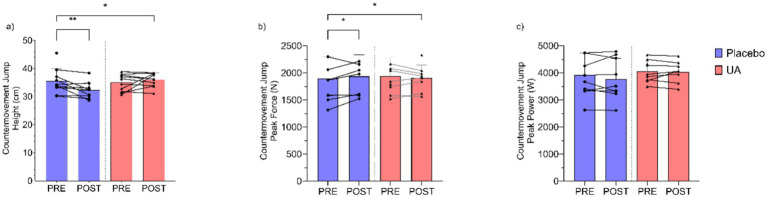
Results for countermovement jump **(a)** height (cm), **(b)** peak force (N), and **(c)** peak power (W) pre and post intervention for Urolithin A (UA) and Placebo groups. Individual data points and lines represent individual players, and error bars denote standard deviation around each mean. *p < 0.05, **p < 0.01.

A significant group-by-time interaction was observed for Countermovement Jump Height (cm) (Figure 2a), with the UA group showing an increase while the placebo group experienced a decrease (*F*(1, 14.90) = 6.828, *p* = 0.020). The estimated improvement (*β*) in CMJ height for the UA group was 3.33 cm (95%CI [0.88, 5.95]) above the change for placebo. A significant interaction was observed for the Countermovement Jump Peak Force (Figure 2b), with the UA group demonstrating a slight reduction while the placebo group demonstrated an increase (*F*(1, 15.13) = 4.722, *p* = 0.046). The estimated difference in the UA group vs. placebo was *β* = −136 N (95%CI [−257, −13]). Finally, Countermovement Jump Peak Power (W) demonstrated no significant group x time interaction effect (*F*(1, 14.95) = 0.069, *p* = 0.797), with minimal changes observed in both the UA and placebo groups (*β* = 25 W, 95%CI [−162, 215]) (Figure 2c).

**Figure 3:**
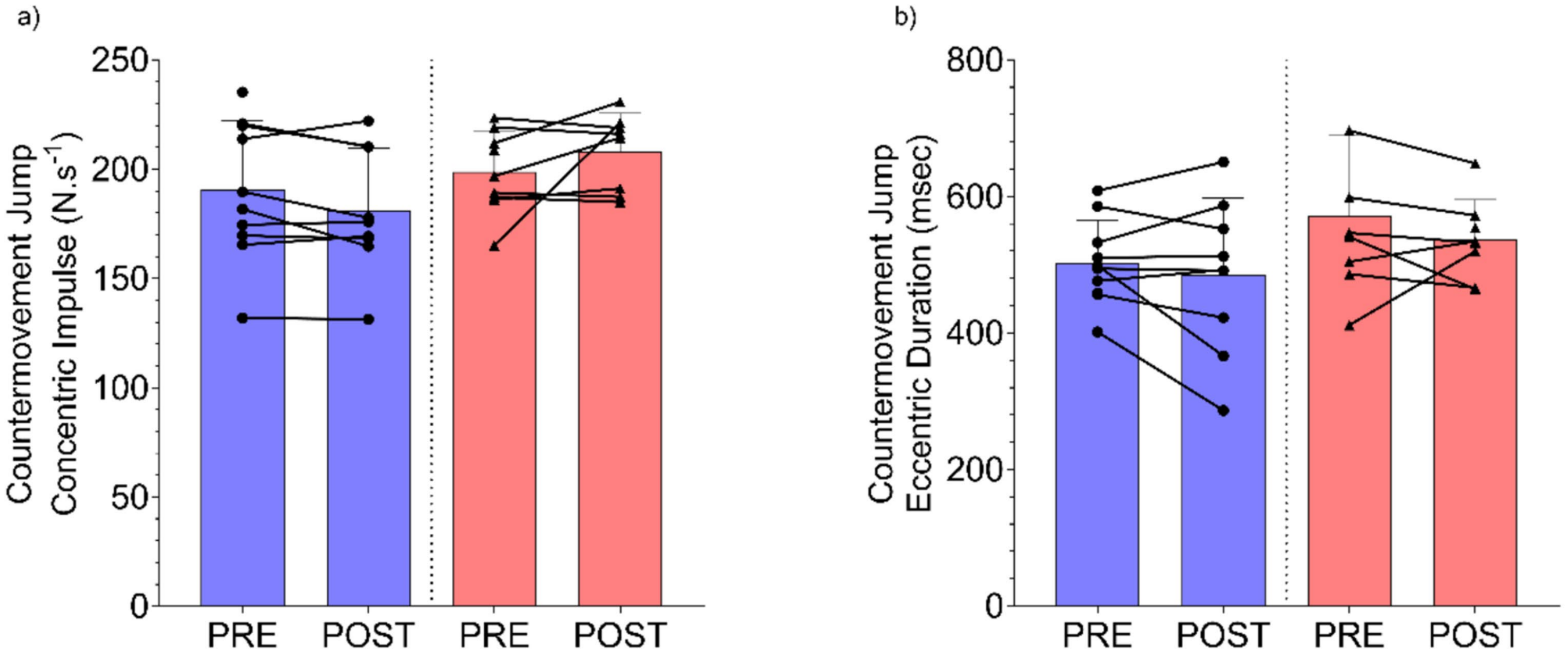
Results for countermovement jump **(a)** concentric impulse (N·s^−1^), and **(b)** eccentric duration (msec) pre and post intervention for Urolithin A (UA) and Placebo groups. Individual data points and lines represent individual players, and error bars denote standard deviation around each mean.

Countermovement Jump Concentric Impulse (N·s^−1^) demonstrated no significant group x time interaction effect (*F*(1, 15.2) = 4.33, *p* = 0.055), with minimal change (*β* = 15.5 N·s^−1^, 95%CI [1.1, 30.2]) observed between the UA and placebo groups (Figure 3a). Finally, there was no group x time interaction (*F*(1, 16.2) = 0.147, *p* = 0.707) between UA and placebo groups for Countermovement Jump Eccentric Duration (msec) (*β* = −15.4 msec, 95%CI [−94.0, 62.9], Figure 3b).

#### Saliva assays

The three measures of antioxidant status (free radical scavenging, hydrogen peroxide scavenging and organic hydroperoxide scavenging) were moderately to moderately-strongly related (*p* < 0.05–0.001) across the entire data set (*R*^2^ = 0.30–0.64; Supplementary Figure 1), indicating that these assays were measuring different facets of antioxidant defence.

**Figure 4:**
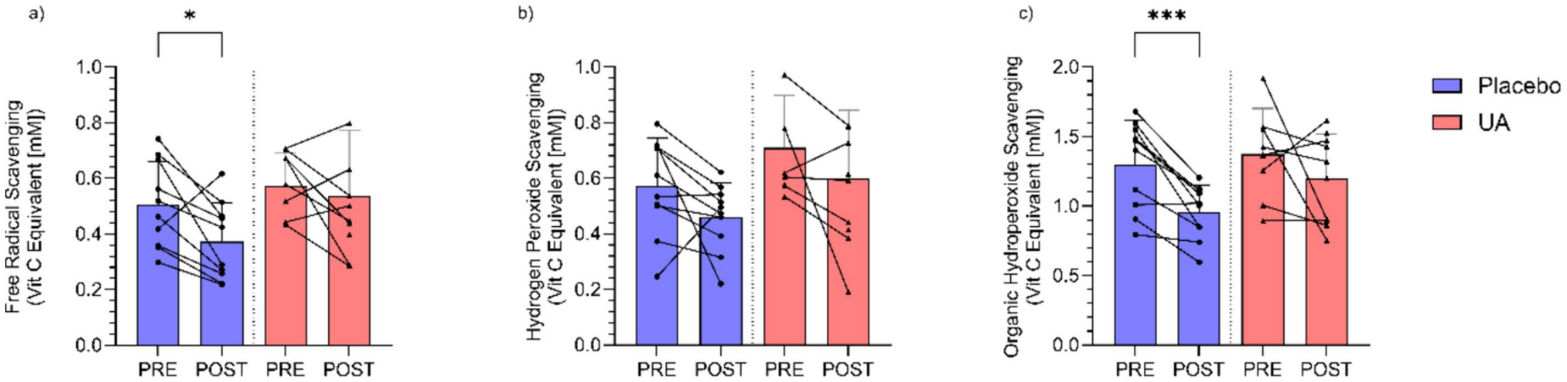
Results for saliva assays for **(a)** free radical scavenging (mM), **(b)** hydrogen peroxide scavenging (mM), and **(c)** organic hydroperoxide scavenging (mM) activity pre and post intervention for Urolithin A (UA) and Placebo groups. Individual data points and lines represent individual players, and error bars denote standard deviation around each mean. *p < 0.05, ***p < 0.001.

There were no overall significant group × time interactions observed for Free Radical Scavenging (Figure 4a) (*F*(1, 17.79) = 0.96, *p* = 0.350), Hydrogen Peroxide Scavenging (Figure 4b) (*F*(1, 34) = 0.003, *p* = 0.997), and Lipid Peroxide Scavenging (Figure 4c) (*F*(1, 17.38) = 0.95, *p* = 0.353). Completion of the intense physical training regime in the absence of UA significantly reduced antioxidant capacity of the athletes from pre- to post-intervention, as seen the in salivary values for free radical scavenging (*p* < 0.05) and organic hydroperoxide scavenging (*p* < 0.001) activities (Figures 3a,c). By contrast, in the UA treatment group, all measures of antioxidant activity showed with no significant differences between time points (Figure 4).

### Dietary intake recall

Analysis of the pre-intervention dietary intake recall data indicated that players consumed an average of 2,437 ± 554 kcal per day. The macronutrient distribution of total energy intake comprised 24.1 ± 6.7% from protein, 39.6 ± 7.9% from carbohydrates, and 33.3 ± 8.3% from fat. Post-intervention dietary analysis revealed that players consumed a similar overall energy consumption with an average of 2,363 ± 507 kcal per day. Macronutrient distribution indicated that 19.9 ± 5.0% of total energy intake was derived from protein, 47.0 ± 7.4% from carbohydrates, and 29.4 ± 5.6% from fat. A summary of this including macronutrient consumption on a per gram basis is presented in Supplementary Table 2.

## Discussion

This pilot study is the first study to examine the effects of Urolithin A (UA) supplementation on performance outcomes in soccer players over a 6 week pre-season training period. Soccer is a physically demanding sport requiring players to perform intermittent bouts of high-intensity activity interspersed with lower-intensity movements (1). The preseason phase is critical for developing these physical capacities through structured and progressively intensified training. In this study, through their pre-season training, players were exposed to substantial weekly workloads, including high-speed running and sprinting. Overall, the findings support the potential use and benefit of UA to enhance aerobic endurance and lower limb performance outputs when used alongside a training intervention. Although overinterpreting these findings should be cautioned as adherence to training and training loads differed somewhat between groups, and there were differences in baseline values between groups. Additionally, the intervention as it was designed and administered in this study demonstrated high feasibility and acceptability among this elite academy soccer athlete cohort. As this is an initial pilot study establishing both the estimated change across these variables and the feasibility and acceptability of the intervention in this population, further and more appropriately powered work is necessary to confirm the estimated effect of the intervention on these and similar performance outcome measures. For example, using the method of Borm et al. (34) in accounting for within participant correlation and the results of this study for the primary outcome measure of YoYo IRT distance, ~34 participants per group would be required to achieve 80% power at a two-sided statistical significance of 0.05.

Through the study, UA supplementation was observed to generate a statistically significant improvement in aerobic endurance, as measured by the Yo-Yo Intermittent Recovery Test Level 1 (Yo-Yo IRT1). Here, UA supplementation alongside their regular training improved YoYo IRT distance by approx. 239 m more than the placebo group. Such a change is greater than the typical error observed in similar aged non-elite and more elite soccer cohort athletes in a test–retest scenario (~77–172 m) (35). This increase suggests that the change has the potential to be practically meaningful although group differences in training effects, baseline values, and adherence could potentially influence this interpretation. Whilst being speculative, these results could potentially also relate to the purported mechanisms of UA in enhanced mitochondrial function and oxidative capacity. These findings may therefore align with previous research potentially indicating UA’s role in promoting mitophagy and improving endurance-related outcomes in older and clinical populations (15, 16, 21), although further mechanistic and confirmatory research would be necessary to examine that. Singh et al. (16) identified clinically meaningful improvements in maximal oxygen consumption (~3 mL/kg/min) and physical performance through a six-minute walk test (~30 m) after administration of 1,000 mg UA for 16 weeks in a cohort of older individuals. When examining the underlying metabolic pathway changes in skeletal muscle through proteomics, UA supplementation was associated with mitochondrial gene upregulation, enrichment changes in proteins related to the Parkin-mediated mitophagy system, and improvements in TCA cycle and oxidative phosphorylation proteins (16). Whilst not quantified in the present study, it’s likely that these underlying physiological processes and mechanisms may, at least in part, explain some of the observed improvements in aerobic endurance capacity. Further work quantifying the metabolic (or metabolomic) changes in different tissues and biofluids (e.g., plasma) would be necessary to confirm if that is an accurate prediction in these athletic populations. It is important to note that dietary intake was not standardised throughout the intervention, and while energy intake was stable, the dietary intake data reveal shifts in macronutrient consumption content to an increasing proportion of carbohydrates and decreasing protein intake from the start to the end of the intervention. The relevance of this to the change in performance outcomes in the present study is unclear but cannot be discounted.

For neuromuscular performance, UA supplementation resulted in a significant increase in countermovement jump (CMJ) height with training, compared to the placebo group. This change was greater than the test–retest typical error (3.3 vs. ~2.0 cm) in a similar academy soccer cohort (36), suggesting a potentially meaningful improvement with UA supplementation. For the CMJ test, peak force was observed to significantly decrease in the UA group following the intervention, and no significant changes were observed for CMJ peak power. Importantly, the peak force change in the present study (~136 N) was similar the typical error that has been observed from test to test in a similar academy soccer cohort (~126 N) (36), making the interpretation of our findings less clear. There is a suggestion that UA supplementation may improve neural aspects of performance adaptations in comparison to the structural force generating and transmitting adaptations (e.g., hypertrophy, tendon stiffness) (37). This suggestion is based on a limited evidence base and requires further research to support or refute. Nonetheless, the peak force results in the present study would seem at odds with those of Singh et al. (16) who observed clinically meaningful and statistically greater improvements in hamstring muscle strength and leg flexion torque with UA supplementation compared to placebo. When Singh et al. (16) and other studies (22) have observed improvements in force output with UA supplementation, they have done so using fixed joint tasks (e.g., knee extension isokinetic dynamometry) while the present study examined force and power output using a dynamic movement task in the CMJ which includes the stretch shortening cycle. The lack of interchangeability in testing between dynamic and more fixed joint tasks is supported by prior research (38). As soccer athletes are required to perform dynamic tasks such as accelerations, decelerations, and jumping in training and match-play, the use of dynamic tasks as in the present study for performance assessment is arguably a more ecologically valid approach.

Across the CMJ variables examined in the present study, whilst there was a non-significant effect, players in the UA group appeared to have modified their jump strategy somewhat to a greater extent than those in the placebo to increase the time on the ground and the resulting concentric impulse to achieve a greater jump height. This would assist in explaining both the lower peak force and the greater jump height observations. Previous work has identified this as a common alteration to jump strategy, particularly when athletes are fatigued (39). These findings may also be a function of the limited sample size in the present study. The mechanistic basis for this finding, and the potential differences in dynamic exercise (i.e., jump) performance with UA supplementation is unclear and may be a function of a small sample size. A larger sample of players would be necessary to explore and confirm or refute these findings in future work. Another factor which may have influenced the findings is the lack of a ‘washout’ period following training but prior to the follow up testing which may have resulted in residual fatigue being present for the players, which was stochastic in nature.

Supplementation with UA is thought to provide physiological benefits across several pathways including by reducing oxidative stress. Despite the purported benefits of UA to bolstering antioxidant defence and reducing ROS (40), there were no significant differences observed between the groups and timepoints in relation to the saliva-based antioxidant assessments. The results of this study revealed that the RoXsta™ system can provide extremely rapid assessments of several different types of antioxidant activity including the capacity to scavenge free radical hydrogen peroxide and organic hydroperoxides that were related [Supplementary Figure 2; Aitken et al. (30)]. Quantitatively, the organic hydroperoxide scavenging activity predominated. Previous studies demonstrating a relationship between intense aerobic exercise and oxidative stress triggered by the intense production of reactive oxygen species by active muscle contraction (9, 41). The enhanced generation of toxic oxygen metabolites during exercise then lowers the levels of total antioxidant protection detected in body fluids such blood (42). In this study, intense physical exercise was found to significantly lower the levels of saliva antioxidant defence seen in the placebo-controlled group but not in the athletes given UA antioxidant supplementation. The relevance of this is unclear and likely requires longer supplementation, mechanistic examination (i.e., metabolomics), and more rigorous dietary controls to examine. The pathways by which UA supplementation could influence jump height, outside of broader improvements to recovery influencing training adaptations, are presently not well characterised, and require further research to examine. To the authors knowledge, this is the first time that salivary measures of antioxidant capacity have been used to track the impact of intense physical exercise on redox homeostasis. The result of this study further suggest that the rapid assessments generated by the RoXsta™ system may be of value in managing oxidative stress in athletes by indicating when antioxidant treatment should commence and, just as importantly, when it should cease, to avoid the risk of over-supplementation and the induction of reductive stress (43).

The intervention as it was delivered in the present study appeared to be tolerated and accepted by the players. This is evidenced by the responses to the feasibility and acceptability questionnaire which was completed by each participant. As the questionnaire responses indicated, the majority of players had positive responses regarding the supplement and found it easy to engage with the intervention. There was also a majority who perceived that they attained improvements in aerobic and power performance through the intervention. Finally, the majority of players also agreed that the intervention was feasible for a larger-scale implementation. Alongside the statistical considerations for powering future research using the results of the present pilot study, these feasibility and acceptability results may also be useful in designing (or co-designing) the intervention in future research in team sport populations (e.g., soccer, rugby league, rugby union).

## Limitations

There are several limitations which should be acknowledged when interpreting our findings. First, the short intervention duration of 6 weeks may not have been sufficient to observe meaningful changes in some performance domains, such as sprint speed or maximal strength, which often require longer training exposure and adaptation. Presently, the manufacturer recommends 8 weeks as the minimum duration to see optimal benefits with the formulation of UA used in this study. Secondly, the relatively small sample size (*n* = 20) of this pilot study also limits statistical power and increases the risk of type II error. As such, our findings should be interpreted with caution until such time as larger and more well powered studies are conducted to identify the influence of UA on performance outcomes. Thirdly, while randomisation allows for the minimisation of certain sources of bias, in this study it did mean that the two groups were not necessarily balanced for the primary outcome at baseline. Whilst balancing covariates at baseline across such a range of variables is not always possible or even desirable (44), and the baseline values were accounted for in the modelling process, there are ceiling effects with the adaptations possible over the study’s duration which must be acknowledged and which may influence the findings. Matching at baseline for the primary outcome (i.e., aerobic endurance capacity) may be a pertinent approach in future research to ameliorate this issue. Whilst the players dietary intake was captured, diet was not standardised either during the intervention nor around the pre- and post-intervention testing occasions. Finally, the study also employed a single blind design, where only participants were blinded to group allocation. This was a practical necessity in this study but may have introduced the potential for researcher bias (either explicit or implicit) during performance testing or data interpretation.

## Future directions

Future research should aim to replicate these findings in larger and more diverse athletic populations. As noted, matching for pre-intervention values for the primary performance outcome may be necessary in such research. In addition, including female athletes and players from different competitive levels, would also be beneficial. Longer intervention periods may also be necessary to capture the physiological adaptations associated with UA supplementation. Additionally, as mentioned in the limitations, using a broader range of biological samples (e.g., bloods) and analysis techniques (e.g., metabolomics) might provide deeper insight into the underlying pathways by which UA is influencing performance. Finally, future studies could also explore the dose–response relationship of UA, as well as the timing of supplementation relative to exercise training, and how this may augment the effect of UA on adaptations.

## Summary

As the first study examining UA supplementation and training adaptation in team sports such as soccer, this study provides novel evidence supporting the use of UA supplementation as a potential ergogenic aid during the preseason phase in sub-elite academy soccer players. The significant improvements in aerobic endurance and CMJ performance in this study suggest UA may enhance mitochondrial efficiency and neuromuscular functions under high training loads. While other variables such as CMJ power and sprint performance did not significantly improve, this may reflect factors related to training specificity and intervention duration. Importantly, high compliance and perceived acceptability reinforce the practical feasibility of using UA in such team sport settings. Though promising, these findings should be interpreted as an initial pilot trial in light of the study’s limitations including its limited six-week duration, and small sample size. As such future research incorporating longer interventions and more mechanistic variables to understand the underlying physiological basis are warranted. Ultimately, UA supplementation could be a viable adjunct to structured training programmes in team sports, particularly when training load is elevated, offering benefits to aerobic and neuromuscular outcomes when applied within a periodised training framework.

## Data Availability

The raw data supporting the conclusions of this article will be made available by the authors without undue reservation.

## Footnotes

^1^ www.sealedenvelope.com
