# Supplementary material for: Effects of Urolithin A supplementation on performance and antioxidant status in academy soccer players during preseason: a pilot randomised controlled trial
Source: Front Nutr. 2025 Oct 30;12:1674446. doi: 10.3389/fnut.2025.1674446 (PMC12611738; doi:10.3389/fnut.2025.1674446)
Supplement: Supplementary file 1 [file Table_1.docx]

**Supplementary Table 1**. Feasibility and acceptability survey results including responses across the different rating scales and the median (± interquartile range [IQR]). The highest percentage response across each question is in bold. The colour coding in the table reflects the differences in description across the 1 – 5 Likert rating scale.

| **Question** | **Response (Percentage)** | | | | | **Median (IQR)** |
| --- | --- | --- | --- | --- | --- | --- |
| How did you feel about the supplement intervention in this study? | Strongly dislike | Dislike | No opinion | Like | Strongly like | 4 (0) |
|  | 0 (0%) | 0 (0%) | 3 (15%) | **13 (65%)** | 4 (20%) |  |
|  | | | | | |  |
| How much effort did it take to engage with the supplement intervention? | Extreme effort | Considerable effort | Moderate effort | Little effort | No effort at all | 4 (1) |
|  | 0 (0%) | 1 (5%) | 2 (10%) | **12 (60%)** | 5 (25%) |  |
|  | | | | | |  |
| The supplement has improved my aerobic capacity (YoYo IRT1 scores) over the preseason period? | Strongly disagree | Disagree | No opinion | Agree | Strongly agree | 4 (1) |
|  | 0 (0%) | 0 (0%) | 7 (35%) | **10 (50%)** | 3 (15%) |  |
| The supplement has improved my force and power (countermovement jump) over the preseason period? | Strongly disagree | Disagree | No opinion | Agree | Strongly agree | 4 (1) |
|  | 0 (0%) | 2 (10%) | 6 (30%) | **11 (55%)** | 1 (5%) |  |
| It’s clear to me how the supplement helps with supporting training adaptations through the preseason? | Strongly disagree | Disagree | No opinion | Agree | Strongly agree | 4 (1) |
|  | 0 (0%) | 0 (0%) | 8 (40%) | **10 (50%)** | 2 (10%) |  |
| Using the supplement in the way it was used in this study would be feasible with a larger group of players in a future study? | Strongly disagree | Disagree | No opinion | Agree | Strongly agree | 4 (1) |
|  | 0 (0%) | 0 (0%) | 3 (15%) | **11 (55%)** | 6 (30%) |  |
| Engaging with the supplement in this study interfered with my other priorities | Strongly disagree | Disagree | No opinion | Agree | Strongly agree | 2 (1) |
|  | 7 (35%) | **9 (45%)** | 3 (15%) | 1 (5%) | 0 (0%) |  |
|  | | | | | |  |
| How acceptable was the supplement intervention in this study to you? | Completely unacceptable | Unacceptable | No opinion | Acceptable | Completely acceptable | 4 (0) |
|  | 0 (0%) | 0 (0%) | 4 (20%) | **12 (60%)** | 4 (20%) |  |
