# Supplementary material for: Effects of Urolithin A supplementation on performance and antioxidant status in academy soccer players during preseason: a pilot randomised controlled trial
Source: Front Nutr. 2025 Oct 30;12:1674446. doi: 10.3389/fnut.2025.1674446 (PMC12611738; doi:10.3389/fnut.2025.1674446)
Supplement: Supplementary file 2 [file Table_2.docx]

**Supplementary Table 2**. Summary of the 24 hour dietary intake recall method pre-intervention (pre) and post-intervention (post) using FoodWorks (Xyris Software, Brisbane, Australia), which utilises the AUSNUT 2011–13 food composition database developed by Food Standards Australia New Zealand (FSANZ) to calculate nutrient intakes.

| Pre | | | | | | | | |
| --- | --- | --- | --- | --- | --- | --- | --- | --- |
|  | **Carbohydrates (g)** | **Protein (g)** | **Total Fats (g)** | **Carbohydrates (%)** | **Protein (%)** | **Total Fats (%)** | **Energy**  **(kJ)** | **Energy (kcal)** |
| Mean | 244.1 | 140.8 | 92.8 | 39.6 | 24.1 | 33.3 | 10194.4 | 2436.5 |
| Standard Deviation | 73.7 | 36.0 | 36.4 | 7.9 | 6.7 | 8.3 | 2317.0 | 553.8 |
| Post | | | | | | | | |
|  | **Carbohydrates (g)** | **Protein (g)** | **Total Fats (g)** | **Carbohydrates (%)** | **Protein (%)** | **Total Fats (%)** | **Energy**  **(kJ)** | **Energy (kcal)** |
| Mean | 277.0 | 117.8 | 80.6 | 47.0 | 19.9 | 29.4 | 9885.5 | 2362.7 |
| Standard Deviation | 55.6 | 43.4 | 25.4 | 7.4 | 5.0 | 5.6 | 2120.3 | 506.8 |
