# Supplementary figures and images for: Effects of Urolithin A supplementation on performance and antioxidant status in academy soccer players during preseason: a pilot randomised controlled trial
Source: Front Nutr. 2025 Oct 30;12:1674446. doi: 10.3389/fnut.2025.1674446 (PMC12611738; doi:10.3389/fnut.2025.1674446)

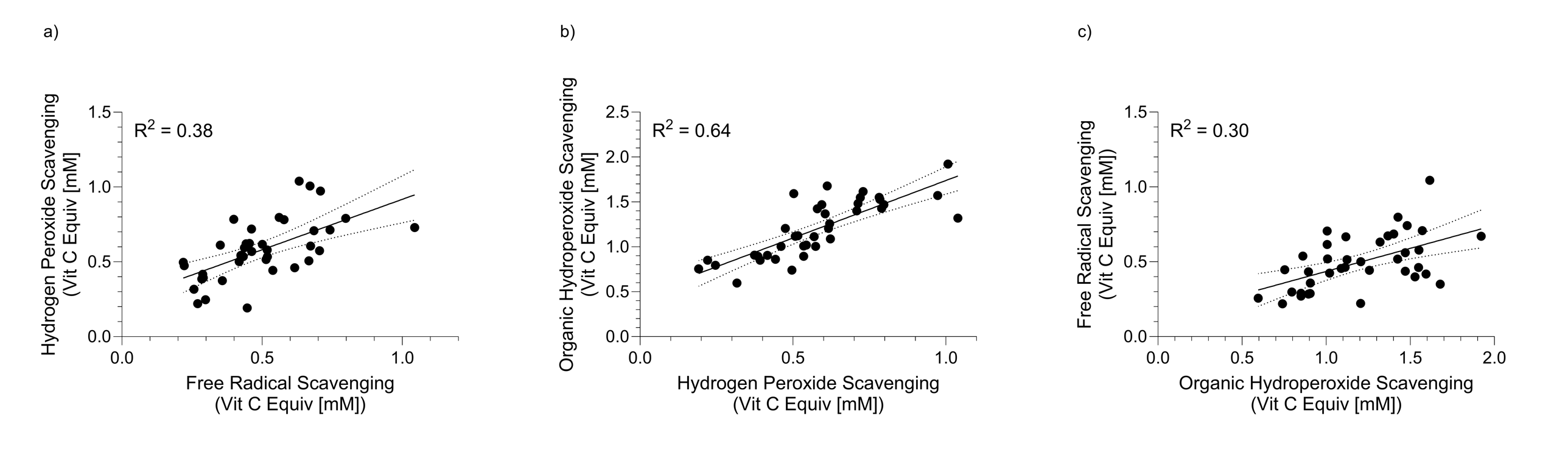

Supplement: Supplementary file 3 [file Image_1.tif]
