# Supplementary material for: Effects of Urolithin A supplementation on performance and antioxidant status in academy soccer players during preseason: a pilot randomised controlled trial
Source: Front Nutr. 2025 Oct 30;12:1674446. doi: 10.3389/fnut.2025.1674446 (PMC12611738; doi:10.3389/fnut.2025.1674446)
Supplement: Supplementary file 4 [file Image_2.pdf]

Figure 1: CONSORT 2025 Flow Diagram

Flow diagram of the progress through the phases of a randomised trial of two groups (that is, enrolment, intervention allocation, follow-up, and data analysis)

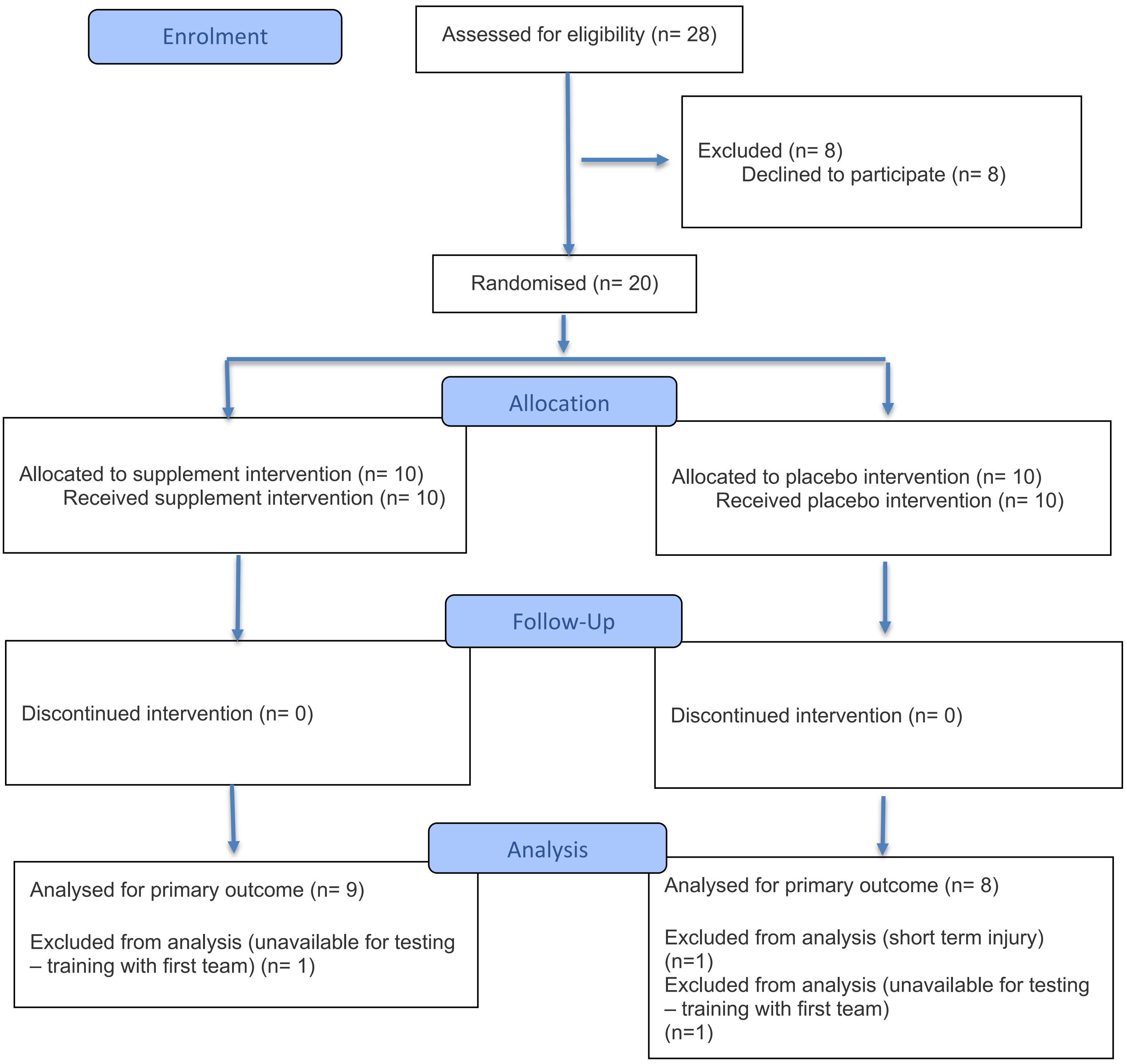

Citation: Hopewell S, Chan AW, Collins GS, Hróbjartsson A, Moher D, Schulz KF, et al. CONSORT 2025 Statement: updated guideline for reporting randomised trials. BMJ. 2025; 388:e081123. https://dx.doi.org/10.1136/bmj-2024-081123
© 2025 Hopewell et al. This is an Open Access article distributed under the terms of the Creative Commons Attribution License (https://creativecommons.org/licenses/by/4.0/), which permits unrestricted use, distribution, and reproduction in any medium, provided the original work is properly cited.
